# What is actually measured in process evaluations for worksite health promotion programs: a systematic review

**DOI:** 10.1186/1471-2458-13-1190

**Published:** 2013-12-17

**Authors:** Debbie Wierenga, Luuk H Engbers, Pepijn Van Empelen, Saskia Duijts, Vincent H Hildebrandt, Willem Van Mechelen

**Affiliations:** 1 Body@Work, Research Centre on Physical Activity, Work and Health, TNO-VUmc, Amsterdam, Netherlands; 2 Department of Public and Occupational Health, EMGO + Institute for Health and Care Research, VU University Medical Centre, Amsterdam, Netherlands; 3 Netherlands Organization for Applied Scientific Research, TNO Expertise Centre Life Style, P.O. Box 2215, 2301, CE Leiden, the Netherlands

**Keywords:** Implementation, Worksite health promotion program, Systematic review, Process evaluation, Lifestyle intervention, Determinants of implementation, Barriers and facilitators

## Abstract

**Background:**

Numerous worksite health promotion program (WHPPs) have been implemented the past years to improve employees’ health and lifestyle (i.e., physical activity, nutrition, smoking, alcohol use and relaxation). Research primarily focused on the effectiveness of these WHPPs. Whereas process evaluations provide essential information necessary to improve large scale implementation across other settings. Therefore, this review aims to: (1) further our understanding of the quality of process evaluations alongside effect evaluations for WHPPs, (2) identify barriers/facilitators affecting implementation, and (3) explore the relationship between effectiveness and the implementation process.

**Methods:**

Pubmed, EMBASE, PsycINFO, and Cochrane (controlled trials) were searched from 2000 to July 2012 for peer-reviewed (randomized) controlled trials published in English reporting on both the effectiveness and the implementation process of a WHPP focusing on physical activity, smoking cessation, alcohol use, healthy diet and/or relaxation at work, targeting employees aged 18-65 years.

**Results:**

Of the 307 effect evaluations identified, twenty-two (7.2%) published an additional process evaluation and were included in this review. The results showed that eight of those studies based their process evaluation on a theoretical framework. The methodological quality of nine process evaluations was good. The most frequently reported process components were dose delivered and dose received. Over 50 different implementation barriers/facilitators were identified. The most frequently reported facilitator was strong management support. Lack of resources was the most frequently reported barrier. Seven studies examined the link between implementation and effectiveness. In general a positive association was found between fidelity, dose and the primary outcome of the program.

**Conclusions:**

Process evaluations are not systematically performed alongside effectiveness studies for WHPPs. The quality of the process evaluations is mostly poor to average, resulting in a lack of systematically measured barriers/facilitators. The narrow focus on implementation makes it difficult to explore the relationship between effectiveness and implementation. Furthermore, the operationalisation of process components varied between studies, indicating a need for consensus about defining and operationalising process components.

## Background

Employees with unhealthy lifestyle behaviors and overweight or obese employees are less productive at work, show a decreased work ability and take more sick days compared to employees with a healthy lifestyle [1-4]. An unhealthy lifestyle can be characterized by one or more of the following behaviors; low physical activity levels, an unhealthy diet, smoking, frequent alcohol use and poor levels of relaxation (i.e. mental health and vitality). Because employed adults spend about half of their workday waking hours at their workplace, the worksite may be an effective setting to increase employees health and productivity [5-8]. Therefore, in the past decade, numerous worksite health promotion programs (WHPPs) have been conducted to improve employees’ health and lifestyle. However, not all of these programs were successful [5-7].

In order to change employees’ health and lifestyle, programs need to work as intended (and therefore avoid theory failure). Additionally, they must be effectively transferred from research to practice and be maintained over time [9]. Programs often fail to reach potential participants adequately due to a lack of adoption, communication, or program sustainability which could lead to low participation levels (in other words, program failure) [10]. Furthermore, research starts to recognize the importance of evaluating different implementation outcomes (such as recruitment, dose delivered, dose received, fidelity, satisfaction, and maintenance) and the contextual factors that hinder or facilitate the implementation of a WHPP [11-14]. These contextual factors are related to characteristics of the context, organization, implementer, program and participants [11,12,15]. Since WHPPs are often comprehensive interventions with multiple components, it is difficult to determine the overall level of implementation and investigate which specific intervention components have been successful [14,16].

A review by Durlak and Dupre (2008) showed that higher levels of implementation improve program outcomes, suggesting that there should be an adequate focus on the implementation process [13]. However, most research focuses primarily on measuring the effects of a WHPP, with an occasional process evaluation performed after implementation. However, systematic process evaluations can produce valuable insights into the interpretation of the (lack of) effects of an intervention by identifying successful and unsuccessful program components, thereby allowing researchers to optimize their program [16-19]. Furthermore, process evaluations can help to identify barriers and/or facilitators influencing the implementation process, while taking into account the different actor levels at which these factors play a role [16,18]. These are valuable outcomes that can be used to improve program implementation in the future and across other settings. Hence, effect evaluations should be accompanied by systematic and real-time process evaluations [20,21].

**Figure 1:**
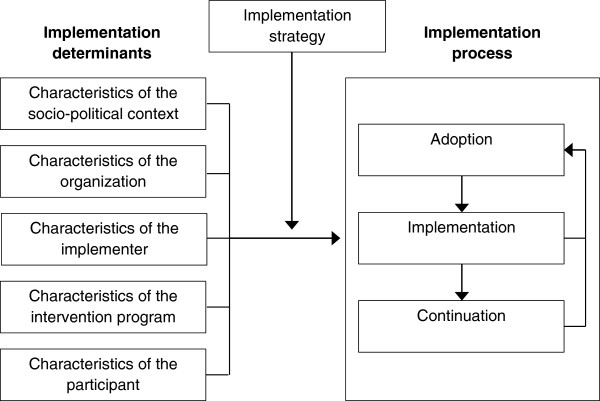
Theoretical framework.

Murta et al. (2007) looked at the quality of process evaluations accompanying controlled trials studying individual based stress management interventions. They showed that process evaluations are often incomplete and not systematically conducted, meaning that they lacked a theoretical framework and were not planned prior to implementation [22]. However, the quality of the process evaluations that were included in this review was insufficient to make sound conclusions in terms of best predictors for successful interventions. Additionally, Murta’s review only focused on evaluations of occupational stress management interventions published between 1977 to 2003. So their conclusion cannot directly be transferred to recent WHPP focusing on other lifestyle behaviors. In their review, Murta also concluded that the framework of Steckler and Linnan proved to be a useful tool to conduct process evaluations. But in this framework little attention has been given to the great variety of contextual factors that can either hinder or facilitate the implementation process. We therefore recently proposed a framework for a systematic and comprehensive process evaluation based on several theoretical frameworks to gain insight into the implementation process (Figure 1) [11,12,14,15,23]. The four main aspects of this framework relate to determinants of implementation that may influence the implementation process, and the implementation process itself (i.e. adoption, implementation and continuation). These four main aspects are operationalized using a combination of the framework of Steckler and Linnan for process evaluations and the RE-AIM framework [14,23]. So, in order to gain insight in the implementation process, eight different process components at three different actor levels (macro-level: organization and management; meso-level: implementer; micro-level: participant) need to be evaluated using a mixed methods approach [11]. Of these eight components, six components focus on implementation (reach, recruitment, dose delivered, dose received, fidelity, and satisfaction), one component (maintenance) on continuation and the eighth component (context) refers to the determinants of implementation. Since many implementation determinants can be identified, context is further defined by categorizing the barriers and/or facilitators into five main categories: 1) characteristics of the socio-political context, 2) characteristics of the organization, 3) characteristics of the implementer, 4) characteristics of the intervention program, 5) characteristics of the participant [11,12,15].

Some reviews have focused on process evaluations and relevant implementation barriers and/or facilitators [10,12,13,22,24,25]. However, these reviews focused on domains other than WHPPs [12,13,24,25], limited the identification of barriers and/or facilitators to those affecting WHPP participation levels [10], or focused only on process evaluations accompanying stress management interventions [22]. To our knowledge, there has been no systematic review that has examined process evaluations for worksite health promotion programs targeting lifestyle change among employees. The aim of this review was therefore to: (1) further our understanding of the quality of process evaluations alongside effect evaluations for worksite health promotion programs (WHPPs), (2) identify barriers/facilitators affecting implementation, and (3) explore the relationship between effectiveness and the implementation process.

## Methods

### Literature search and study selection

For this systematic review, peer-reviewed studies were eligible for inclusion when they reported an effect evaluation as well as a process evaluation for a worksite health promotion intervention focusing on stimulating a healthy lifestyle (physical activity, nutrition, smoking, alcohol use and relaxation) published in English between 2000 and July 2012. The literature search was conducted in two steps.

Step 1: a literature search was in the online databases Pubmed, EMBASE, PsycINFO, and the Cochrane Central Register of Controlled trials for peer-reviewed WHPP effect evaluations published in English from 2000 to July 2012. Searches included the following combination of keywords: (Randomized controlled trial OR controlled trial) AND (worker* OR employee* OR worksite OR work environment) AND (worksite health promotion OR lifestyle intervention) AND (absenteeism OR sickness absence OR body mass index OR lifestyle OR health behavior OR cholesterol level). To ensure no studies were overlooked this search string was repeated in each database with the addition of the following keywords for each lifestyle behavior separately: ‘physical activity’, ‘smoking’, ‘alcohol use’, ‘healthy diet’ and ‘relaxation’. Since we were only interested in studies focusing on change in actual behavior rather than change in attitude, social norm or self-efficacy keywords were added on outcome measures related to actual behavior change. All the keywords were specified for each database using Mesh or Thesaurus terms. The complete search strategy for each database and lifestyle behavior are presented in Additional file 1. The inclusion criteria for the first stage of the selection process were: (1) a randomized controlled (RCT) or controlled trial (CT), (2) an evaluation of the effects of worksite health promotion interventions focusing on physical activity, smoking cessation, alcohol use, healthy diet and/or relaxation at work, and (3) targeting employees aged 18-65 years. With respect to the second inclusion criteria it should be noted that studies investigating interventions that primarily aim to promote a healthy lifestyle as well as interventions that primarily aim to prevent musculoskeletal disorders of which the intervention contains one of the lifestyle components mentioned above were included. The first author (DW) performed an initial selection based on the titles and abstracts of all papers reporting on effect evaluation. The abstracts of the effect evaluations were presented to the fourth author (SD), who was blinded for authors, affiliations, journal and year of publication. In a consensus meeting between both authors (DW and SD), a final selection of effects evaluations was made. When an abstract contained insufficient information or when the authors disagreed, the full paper was retrieved and read. When disagreement persisted, the second author (LE) was asked to decide about eligibility.

Step 2: DW checked whether the selected effect evaluations were accompanied by a peer-reviewed process evaluation. The inclusion criteria for the second stage of the selection procedure were: (1) published in English between 2000 and July 2012, and (2) reporting on implementation/process outcomes. DW first contacted the corresponding authors by e-mail to ask whether the effect evaluation had been accompanied by a paper on implementation/process outcomes. If the corresponding author did not respond or could not provide the requested information, DW first checked the full text paper of the effect evaluation for references to a related process evaluation (in the reference list and/or text). If nothing was found, DW searched all four databases using the title of the study (if known), trial registration number and the names of all authors for an additional formative, implementation or process evaluation. If based on title and abstract it was unclear whether the paper included information on the implementation process of the intervention, the full text paper was retrieved and read.

The full papers were retrieved for all eligible effect evaluation papers paired with a published process evaluation paper. These complete studies were independently assessed for eligibility by the first three authors (DW, LE and PE) before being included in the review. During this final round, LE and PE were blinded for authors, affiliations, journal and year of publication. All references used in eligible effect evaluations and process evaluations were checked by the first author (DW) for other relevant publications that might have been missed in the electronic search (‘snow ball’ procedure).

### Methodological quality assessment

In order to answer the first research question, a methodological quality assessment was performed. The methodological quality of all papers included in the review was independently assessed by the first three authors (DW, LE and PE) using a checklist (Table 1). Disagreements between reviewers were discussed and resolved during consensus meetings.

**Table 1:** Criteria list for the methodological quality assessment of the studies and definitions of the criteria

**Effect evaluations**		
	**Internal validity**/**study design**	
**V1**	Randomization procedure	Positive if a random (unpredictable) assignment procedure sequence of subjects to the study groups was used and if there was a clear description of the procedure and adequate performance of the randomization
**V2**	Similarity of companies	Positive if they controlled for variability in included companies
**V3**	Similarity of study groups	Positive if the study groups were similar at the beginning of the study
**V4**	Dropout	Positive if the percentage of dropouts during the study period did not exceed 20% for short-term follow-up (≤ 3 months) or 30% for long-term follow-up (> 3 months) and adequately described
**V5**	Timing of outcome measurement	Positive if timing of outcome assessment was identical for intervention and control groups and for all important outcomes assessments.
**V6**	Blinding	Positive if the person performing the assessments was blinded to the group assignment
**V7**	Co-interventions	Positive if co-interventions were avoided or comparable.
**V8**	Outcome	Positive when data on outcome was selected with standardized methods of acceptable quality
	**Descriptive criteria**	
**D1**	Eligibility criteria (in- and exclusion criteria)	Positive if in- and exclusion criteria of participants were specified
**D2**	Baseline characteristics	Positive if an adequate description of the study groups was given for demographic variables: gender, age, type of work, hours a week working, education level, baseline main outcome measures
**D3**	Company characteristics	Positive if an adequate description of the included companies was given (type of industry, organizational characteristics)
**D4**	Intervention	Positive if an adequate description was given of the interventions(s): number of intervention aspects, type of interventions, frequency of sessions, intensity of intervention(s)
**D5**	Follow-up	Positive if a follow-up of 6 months or longer was described.
	**Analysis**	
**A1**	Sample size	Positive if an adequate sample size calculation was described
**A2**	Confounders	Positive if the analysis controlled for potential confounders
**A3**	Intention to treat	Positive if the intervention and control subjects were analyzed according to the group belonging to their initial assignment, irrespective of non-compliance and co-interventions.
**Process evaluations**		
**T1**	Model used for evaluation	Positive if a theoretical framework for the evaluation was used and adequately described.
**T2**	Level of evaluation	Positive if implementation was evaluated on 2 or more levels (i.e. macro, meso, micro)
**T3**	Definition of outcome measure (process components)	Positive if the definition of the outcome measures (process variables and barriers and/or facilitators) were accurately described
**T4**	Reported process variables	a. Positive if four or more process evaluation variables are evaluated (in process evaluation)
		b. Positive if barriers or facilitators on 1 or more levels are presented
**T5**	Data collection	Positive if 2 or more techniques for data collection were used (triangulation).
**T6**	Timing of data collection	Positive if measurements of barriers and/or facilitators were performed pre-, during and after implementation.
**T7**	Quantitative outcome measures	Positive if data on quantitative outcome was selected with methods of acceptable quality and data on multiple process components was measured.
**T8**	Qualitative data	a. Positive if study design for qualitative data (theoretical framework, participant selection, setting, data collection) were adequately described
		b. Positive if qualitative data was analyzed by two researchers.
**T9**	Outcome related to implementation of intervention	Positive if outcomes (barriers and/or facilitators) are related to the quality of implementation

The criteria for the assessment of the included effect evaluations were based on the methodological guidelines for systematic reviews developed by the Cochrane Back Review Group [26]. These guidelines were developed for RCTs studying low back pain. Some Cochrane criteria were therefore omitted or adapted to fit the studies included in this review. Other reviews focusing on worksite interventions have used an adapted version of this guideline [5,27]. The final criteria list consisted of three main categories: internal validity (n = 8), descriptive criteria (n = 5) and analysis (n = 3). Items were scored as positive (+), unsatisfactory (+/-) or negative (-). If an item was not applicable, this was stated. The quality of studies was considered to be ‘above average’ if the overall validity score (V) was above 50%, and a score above 75% was considered to represent relatively good quality [26,28]. The overall validity score (V) was based on the eight internal validity criteria (V1-V8). When items were not applicable (N/A), they were not included in the percentages.

Given the absence of a standardized assessment form, we defined our own criteria for the methodological quality assessment of process evaluations on the basis of our proposed conceptual framework, shortly explained in the introduction and published elsewhere [11]. These criteria are described in detail in Table 1. The final criteria list included nine items. Items were scored as positive (+), unsatisfactory (+/-) or negative (-). If an item was not applicable, this was stated. The quality of studies was considered to be ‘above average’ if the overall validity score (T) was above 50%, and a score above 75% was considered to represent relatively good quality. This validity score (T) was based on the nine internal validity criteria (T1-T9). Non-applicable items (N/A) were not included in the percentages.

### Data extraction

The first author (DW) extracted the data using a predefined format. For the effect evaluations, information was extracted about study design, company type, study population, intervention content, and intervention goal. In addition, although not the main aim of this review, the proportion of affected primary outcomes was extracted to provide some information about the effectiveness of the program. For the process evaluations information was extracted about data collection, methods, timing of measurements, level of evaluation, type of evaluation, linking effect to implementation outcomes, model for process evaluation, reported process components and reported barriers and/or facilitators. During data extraction, the process components reported in the studies were classified under the eight process components (recruitment, reach, dose delivered, dose received, fidelity, satisfaction and maintenance, and context) on the basis of the definitions proposed by Wierenga et al. [11,14]. If a study used another framework/model with additional components, these were classified separately. The barriers and/or facilitators found in the studies were assigned to five categories based on the review by Fleuren et al. (2004): socio-political context, organization, implementer, program and participants [12,15]. When something was unclear, advise was asked from the second (LE) and third author (PE). The complete data extraction form can be obtained from the corresponding author.

## Results

### Study selection

**Figure 2:**
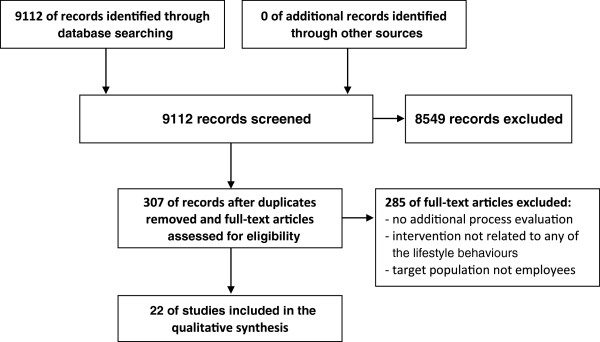
Flowchart of study selection process.

The initial computerized search identified 9112 articles (see flow chart in Figure 2). Initial screening of the titles and abstracts produced 704 potentially relevant articles looking at all lifestyle behaviors. 8408 articles were excluded because they were not (R) CT related to a WHPP with the aim of changing employee lifestyles. We excluded 397 of the 704 potentially relevant articles on the grounds that articles were found in multiple databases (Pubmed, EMBASE, PsycINFO, Cochrane Central Register of Controlled Trials), or in the different searches based on lifestyle behavior. Interventions not related to any of the five health behaviors or not targeting employees were also excluded.

A total of 307 full-text articles were retrieved and checked for related process evaluation articles, resulting in the exclusion of another 277 articles. The main reasons for exclusion were: no additional published process evaluation for the same study, not targeting employees, or no lifestyle component. Thirty studies reporting on both the effects and the implementation process of the same WHPP were checked for eligibility. After reading the papers, eight studies were excluded because, contrary to expectations, they did not perform a process evaluation. No articles were added after snowballing the references in the selected studies. Finally, twenty-two studies met the selection criteria and were included in this review (Additional file 2).

### Intervention and study characteristics

The general study characteristics are presented in Additional file 2; second column, in order to give an overview of the type of studies included. Half of the studies were conducted in the USA [29-39], and the other half in Europe [40-50], mostly in the Netherlands [40,41,45,47-49]. Nineteen (86%) studies used a randomized controlled trial design [29,30,33-36,38-50] and three (14%) a controlled trial design [31,32,37]. Fourteen (63%) studies reported on interventions targeting physical activity (PA) [29-34,40-44,48-50]. Two of these studies focused mainly on preventing musculoskeletal disorders [40,44], but the intervention included a PA component and so these studies were included in the review. Twelve studies (55%) reported on the effect and process evaluation of interventions targeting healthy nutrition [29,32-37,41,45,47-49]. Furthermore, five (23%) studies reported interventions for smoking and tobacco use [36-39,41], and two (9%) studies focused on relaxation [46,48]. None of the twenty-two studies targeted alcohol use.

### Methodological quality assessment

Table 2 shows the methodological quality scores of the included studies. Initial disagreement between the reviewers about 50 of the 448 effect evaluation criteria (11%) and 46 of the 207 process evaluation criteria (22%) were mainly attributable to the interpretation of the items. The differences concentrated primarily on drop-out (V4), company characteristics (D3), intention to treat (A3), data collection for process evaluation (T5), and the timing of data collection for process evaluation (T6). Full agreement was reached after two discussion sessions, thereby completing the scoring process.

**Table 2:** Overall scores of the methodological quality of the included studies

**First author (Year)**	**Methodological quality assessment criterion – effect evaluations**									**Methodological quality assessment criterion – process evaluation**									
	**V1**	**V2**	**V3**	**V4**	**V5**	**V6**	**V7**	**V8**	**Validity score (V) in %**	**T1**	**T2**	**T3**	**T4**	**T5**	**T6**	**T7**	**T8**	**T9**	**Validity score (T) in %**
Driessen et al. (2010, 2011)**	+	+	+/-	+/-	+	+	+	+	87,5	+	+	+	+	+/-	+	+	+	+	94,4
Groeneveld et al. (2010, 2011)*	+	N/A	+	+/-	+	+/-	N/A	+	83,3	-	+	+	+	-	+	+	N/A	+	75
French et al. (2010)*	+/-	+	+/-	N/A	+	N/A	+/-	+	75	-	N/A	-	-	-	+/-	+/-	N/A	-	14,3
Dishman & Wilson et al. (2009, 2010)	+	+/-	+/-	-	+	-	+/-	+	56,25	+	+	+	+	+	+	+	+	+	100
Yap et al. (2009, 2010)*	N/A	N/A	+	+	+	N/A	N/A	+	100	-	-	-	-	-	-	N/A	+	-	12,5
Gilson et al. (2007, 2008)	+	N/A	+	-	+/-	-	-	+	50	-	-	+	-	-	+/-	N/A	+/-	-	25
Goetzel, DeJoy, Wilson et al. (2007, 2009-2011)^a^ ***	N/A	+/-	+/-	-	+	N/A	+/-	+	75	+	+	+	+/-	+	+	+	+	-	83,3
Lemon, Estabrook et al. (2010-2011)	+/-	+	+/-	+	+	-	-	+	62,5	+	+	+	+	+	+	+	+	+	100
Andersen et al. (2011)	+	N/A	+	+	+	+	+	+	100	-	-	+/-	-	-	-	+	N/A	+	31,25
Haukka, Pehkonen et al. (2009,2010)	+	-	+	+	+	N/A	-	+	71,4	-	+	+	+	+/-	+	+	+	+	83,3
Sorensen, Hunt et al. (2005, 2007)	-	+	+	+/-	+/-	-	-	+	50	-	+	+	+	-	+	+	+	-	66,67
Beresford et al. (2000, 2001, 2010)*	-	+	+	-	+	-	-	+	50	-	+	+	+/-	+/-	+	+	N/A	+	75
Sorensen, Hunt et al. (2007, 2010)	-	N/A	+	+	+	-	-	+/-	50	-	-	+/-	-	-	+/-	+/-	N/A	+/-	25
Steenhuis et al. (2004)	-	+/-	+/-	-	+	-	-	+	37,5	-	-	-	+/-	-	-	N/A	-	+/-	12,5
Sorenson, Quintiliani et al. (2010)	_	+	+	+	+/-	-	-	+	56,25	-	+	+	-	-	+	+	N/A	+	62,5
Stoddard, Hunt et al. (2003, 2005)	-	+	+	N/A	+	-	-	+/-	50	-	+	+/-	+/-	-	+	+	N/A	-	50
Volpp, Kim et al. (2009, 2011)	+	-	+	+	+	-	-	+	62,5	+/-	-	+/-	-	+/-	+	+	+	+	61,1
Hasson et al. (2005, 2010)	+	-	+	+	+	-	-	+	62,5	+	-	+	-	+	+	+	N/A	+/-	68,75
Vermeer et al. (2011)	+/-	-	-	+/-	+/-	-	-	+	31,25	+	+	+	+	+	+	+	+	-	88,89
Strijk et al. (2011, 2012)	+	+	+	+/-	+	+	+	+	93,75	+	+	+	+	+/-	+	+	N/A	-	81,25
Verweij et al. (2011, 2012)	+	-	+	+	+	+	+	+	87,5	+	+	+	+	+	+	+	+	+	100
Jorgensen et al. (2011, 2012)	+	-	+	-	+	+/-	+	+	68,75	+	-	+	+	+/-	-	+	N/A	-	56,25

^a^Controlled trial.

*two effect articles scored together.

**two process evaluations scored together.

***three effect articles scored together and 2 implementation articles scored together.

*N*/*A*, not applicable. +, positive. +/-, not sufficient; -, negative. All trials are randomized trials except for the trials indicated with a superscript ‘a’. The maximum score for methodological quality of effect evaluations is 8 (based on validity section V1-V8). The maximum score for methodological quality of process evaluation is 9 (based on section T1-T9).

The methodological quality scores for the effect evaluations of the studies ranged from 37.5% to 100%. Fifteen (68%) effect evaluations were considered to be ‘above average’ (>50%) [29-33,37,39-41,43,44,46,48-50]. Only six of these effect evaluations (27%) were relatively good (quality score >75%) [31,40,41,43,48,49]. The quality scores for the process evaluations ranged from 12.5% to 100%. Eight process evaluations (36%) were relatively good (quality score >75%) [51-58].

Only three (14%) studies scored relatively good (quality score >75%) on both the process as well as the effect evaluation with respect to their methodological quality [51,57,58]. In four (18.2%) studies the methodological quality of both the effect as well as the process evaluation was poor (quality score 50% or less) [36,38,42,45]. No relation was observed between the quality of effect evaluations and process evaluations.

### Process evaluation design

The characteristics of the included process evaluations are presented in Additional file 2; third and fourth column. Most studies (50%) used a mixed methods approach (qualitative and quantitative) to look at the implementation process [51-56,58-62]. With regard to the number of measurements, ten (45%) studies conducted a post-process evaluation only [29,55,59,61-67]. Three (14%) studies collected process evaluation information at three points in time: before, during and after the intervention [54,56,60].

Only two (9%) studies evaluated the implementation process at all three actor levels (the macro-, meso- and micro-levels, see above) [53,60]. The majority (50%) of the studies evaluated the implementation process at both the meso- and micro-levels [51,52,54-59,61,67,68]. A minority of the studies (n = 8; 36%) reported using a theoretical framework to guide their process evaluation [47,51-53,57,58,60,69]. Of these eight studies, four used the framework of Steckler and Linnan (or an adapted version of that framework) [51,57,58,69], and the remaining four studies applied either the integrative model [53], the RE-AIM model [60], a model based on Durlak and Dupre [52], or a model based on Baranowski and Stables, in combination with Rogers’ framework for the diffusion of innovations [56]. It should be noted that the integrative model is originally a model for the development of workplace environmental interventions and not specifically for process evaluations [53].

### Reporting of process evaluation components

The predetermined process components that were measured are presented in Additional file 2; fourth column. Seven of these components (reach, recruitment, dose delivered, dose received, fidelity, satisfaction and maintenance) measure the degree of implementation. The eighth component, context, maps the barriers and/or facilitators that affect implementation. The average number of process evaluation components found in the studies was 3.9, ranging from 1 to 8. Ten (45%) studies evaluated fewer than four process components [29,53,62-64,66-68,70,71]. Six (27.5%) studies reported five or more process components [51,56-58,60,69]. The studies focused mainly on dose received (82%) [29,51,52,54-63,65,67-69,71], dose delivered (68%) [29,51,52,54,55,57-62,65,67],[68], and context (68%) [52,54-57,60,61,64,66,69-74]. Fewer than half of the studies (41%) looked at fidelity [51,52,56-60,65]. Nine (41%) studies measured satisfaction [51,54,57-59,63-66]. Relatively few studies reported on reach (n = 7; 32%) [51,55,57,58,60,61], or recruitment (n = 5; 23%) [56,58,60,64,69]. In addition, two (9%) studies looked at maintenance [56,60].

### Implementation barriers and/or facilitators

Next, we identified barriers and/or facilitators which can affect implementation. However, the barriers and/or facilitators were often not systematically measured by means of questionnaires or interviews and often only observed and documented on the basis of the researchers’ experience. Nevertheless, fifty-four different barriers and/or facilitators were obtained from nineteen (86%) studies (Table 3) [52,54-57,59-64,66,68-74]. On average, the studies described 6.5 [range 1-22] barriers and/or facilitators. None of the studies reported a context analysis prior to implementation. The majority of barriers and facilitators in the five categories as a whole were reported only in a single study.

**Table 3:** **Reported barriers** (**B**) **and**/**or facilitators** (**F**) **in the studies included in this review**

**Main categories**	**Description of the determinants for implementation**	**B**/**F**
*Characteristics of the socio*-*political context*	1. *Compatibility of program with societal developments* (attention for health in society) [74]	F
	2. *Competitive business environment*[53]	B
*Characteristics of the organization*	3. *Organizational reorganization*: reorganization due to take over by another company [68]	B
	4. *Lack of resources*: financial, personnel, material (e.g., equipment, facilities) resources or lack of space or facilities [54,57,66,72,74]	B
	5. *Organizational culture*:	
	(a) Senior leaders emphasized the need to implement the intervention keeping the organizational culture in mind [53]	F
	(b) Intervention did not fit the organizational culture [55,72]	B
	(c) The organizational culture emphasized goal setting and tracks progress towards achieving goals [53]	F
	(d) Worksite culture supported social interaction among workers and between workers and managers [55]	F
	6. *Organizational size*:	
	(a) In a large organization (1000+ employees) there were numerous competing priorities and it was challenging to maintain visibility [60]	B
	(b) In a small organization (<500 employees) it is challenging to assemble a critical mass of potential participants for participation in the intervention [60]	B
	(c) Small organizations tend to receive more intervention components per employee than larger organizations [61]	F
	7. *Amount of company locations*: Different company locations at which the intervention needs to be delivered [74]	B
	8. *Organization*’*s awareness* of perceived benefits of investment [74], and awareness of relevance and economics of health and employee wellness [53,60]	F
	9. *Company image*: the program gives the organization a positive image since it shows that the organization cares about their employees [66]	F
	10. *Perceived responsibility* of employer towards workers health and wellbeing [74]	F
	11. High *staff turnover rate* among employees made it difficult to provide adequate exposure to the intervention [68,69]	B
	12. Good *collaboration* between persons/ structures/ services/ collaborative partners within or outside departments and organizations [54,66,72]	F
	13. *Conflicting relationship between management and researchers*[68]	B
	14. General good *organizational support* for health promotion [53]	F
	15. *Poor psychosocial work environment* consisting of the following the subcomponents: influence at work, work pace quantitative work demands, interpersonal relations [70]	B
	16. *History of social interaction*: Worksite has a history of bringing employees together for social activities and a history of positive social interaction between worker and management [55]	F
	17. *Management support*:	
	(a) Strong (upper) management support for intervention and general health promotion efforts at the organization [55,60,68,72,73]	F
	(b) Unbalanced management support for intervention [55,68]	B
	(c) Managers encouraging workers to attend intervention [55]	F
	(d) Experienced management support are different for junior employees and senior employees [64]	B
	(e) formal approval of upper management before start of intervention [57]	F
	(f) Lack of perceived management support by implementers on site [74]	B
	(h) Management commitment and willingness to provide employees with release time from their usual duties to attend intervention [55]	F
	18. *Management participation and engagement*:	
	(a) Active management participation and involvement alongside and with workers [55,73]	F
	(b) Active management engagement in planning [55]	F
	19. *Relationship between management and employees*: Respectful relationship between management and worker [55]	
*Characteristics of the implementer*	20. *Job position of implementer*: [74]	
	(a) Self-employed (advantage of managing his or her own time)	F
	(b) Internal position (facilitating in scheduling appointments)	F
	(c) external position	
	21. (*Perceived*) *Support for implementers*: [74]	B
	(a) Poor support from co- implementers	B
	(b) Support for implementers to change their routines (applicable when implementer is an occupational physician) [74]	F
	22. *Collaboration between implementers*: lack of possibility to exchange experiences between implementers [74]	B
	23. *Available time of implementer*:	
	(a) Sufficient time available to implement intervention [56,66,72,74]	F
	(b) The intervention involved extra work on top of the heavy workload of the regular duties of the implementer [66]	B
	(c) planning difficulties of implementers with planning al contacts in the intervention period [59]	B
	24. *Expectations of implementer*: implementers expectations were met [74]	F
	25. *Absence of a project leader*/ *leading person*/ *ambassador*[72]	B
	26. *Implementers*’ *compliance* with intervention protocol [52]	F
	27. *Staff turnover among implementers*: drop out of implementers (without replacing them) [69,72]	B
	28. *Absence of decision maker among implementers*: among the implementers there lacked a person who was entitled to make decision at department level [72]	B
	29. *High perceived Level of control* for intervention delivery by provider/implementer [60]	F
	30. *Low level of engagement of implementers* in planning, promoting and providing feedback on intervention activities [55]	B
	31. *Personnel characteristics of implementer*: sufficient skills, knowledge and competence to implement guideline or intervention correctly [55,59,74]	F
*Characteristics of the intervention program*	32. *Degree of* rewards: either financial reimbursement or other incentives [53,68]	F
	33. *Compatibility and alignment* of intervention with:	
	(a) organizations mission statement/business goals/ institutional policy change [53,60,68,74]	F
	(b) policy, culture, norms and current practices of organization [56,58,66,72]	F
	(c) Ease of integration of intervention in working live [64]	F
	34. The intervention fit implementers current work [74]	F
	35. *Intervention is part of the worksites integral health* policy and seen as a pilot for future health promotion policy instead of independent project [57]	F
	36. *Relative advantage*: intervention is advantageous compared to the current situation and no negative consequences were observed and the company, managers, implementers and participants benefit from participation [54,56,66,72,74]	F
	37. *Time*: Project took more time than expected due to high workload of administration and planning [74]	B
	38. *Complexity*: Intervention was not too difficult or complex to implement and execute [56,59,72,74]	F
	39. *Observability* of positive results of the intervention [74]	F
	40. *Risk and uncertainty level*/*Triability*: the degree to which an innovation can be adopted/implemented with minimal risk [56]	F
	41. *Conflicting interest* between worksite and intervention [66]	B
	42. *Timing of intervention activities*: intervention activities coincide with scheduled breaks [68]	F
	43. *Technical problems* (e.g., equipment breaks down) [54,69]	B
	44. *Degree of incorporation* of program communication and interventions into already established communication channels or existing worksite events/meetings [53,55]	F
	45. *Presence of advisory board*: *w*ell-functioning advisory board [55]	F
	46. *Ease of access to the program* by bringing the program to participants and making participation free or inexpensive [53]	F
*Characteristics of the participant*	47. *Needs of participants*:	
	(a) Positive personal preferences for program [63]	F
	(b) No need for intervention (e.g., already being healthy) [74]	B
	(c) Positive program expectation [71]	F
	(d) Prior failed attempts to maintain a healthy lifestyle [62]	B
	48. *Current workload and work structure*/*schedules*: volume of daily tasks, overtime work, shift work, part-time work, irregular work schedules, shifts of different lengths, time-pressures [53,60,64,68]	B
	49. *Work demands*: Workers were unable to participate since they could not leave their work due to work demands, obligations and limited free time and flexibility to leave immediate work area [55,57,60,64]	B
	50. *Time constraints of participants*: lack of time, time constraints and willingness to make time to participate at work [53,54,57,62,63,74]	B
	51. *Amount of peer leaders*: Few peer leaders due to geographically separated worksites made it difficult to establish group cohesion [68]	B
	52. *Lack of social* s*upport*:	
	(a) No interaction with the entire workforce to build worksite-wide social norms and social support) [68]	B
	(b) *Peer support*: difficult to engage in behavior not considered normal by peers [64]	B
	53. *Lack of motivation* of workers to participate in intervention [54]	B
	54. *Participants self*-*efficacy*: Low to medium self-efficacy is a barrier for participation [70]	B

Fewest barriers and/or facilitators were reported in the category ‘characteristics of the socio-political context’. Only two (9%) studies reported a barrier or facilitator in this category (these studies are listed in Table 3). The largest number of barriers and/or facilitators (n = 17) were found in the category ‘characteristics of the organization’. Management support was found most frequently, having been mentioned in eight studies (36%). Strong management support was described as a facilitator, whereas unbalanced or lack of management support was found to be a barrier. The definition of management support varied widely. Another frequently reported barrier was the lack of financial, staffing or material resources (n = 5 studies; 26%). In the category ‘characteristics of the intervention’ fifteen barriers and/or facilitators were identified, with ‘compatibility of intervention with the organization’ being the most commonly reported facilitator in eight studies (42%). The facilitator ‘relative advantage of the intervention’ was reported in five (26%) studies. In the category ‘characteristics of the implementer’ twelve barriers and/or facilitators were identified; the factor ‘available time of the implementer’ was most commonly reported (n = 5; 26%). In the category ‘characteristics of the user’, eight barriers and/or facilitators were identified with ‘time constraints’ was frequently reported as a barrier to participation. Furthermore, we found that high work demands and a high workload were also a barrier to participation.

### Degree of implementation and program effectiveness

Only seven (31.8%) studies evaluated the association between implementation and program outcomes [52,58-62,67]. These studies generally found that the level of implementation in terms of high fidelity and dose was positively associated with a positive change in their primary outcome measures (body weight, waist circumference, Body Mass Index, fruit and vegetable intake, physical activity levels, smoking cessation). This was analyzed by means of linear or logistic regression analysis [52,58-62,67], latent growth modeling [52], dose or as-treated analysis [52,58-62,67], analysis of variance [52,58-62,67], mixed model logistic regression analysis or linear mixed model regression analysis [52,58-62,67], chi square tests [52,58-62,67], and multilevel linear regression analysis [52,58-62,67]. Of the seven studies, three studies found that higher participation levels (dose received) significantly and positively impacted their primary outcome measures (body weight, waist circumference, physical activity levels and smoking cessation) [52,58,62].

In the study on personal and team goal-setting with the aim of increasing leisure-time physical activity, Dishman and Wilson found that high implementation groups had a greater increase in vigorous physical activity over the three time points than did the low implementation sites. For dose received no significant change over time was observed for any of the outcome measures (walking, moderate physical activity, vigorous physical activity) [52]. The results of the study of Volpp and Kim on improving smoking cessation rates by offering financial incentives for smoking cessation, the results showed that the attendance levels to the cessation programs were higher among quitters than among non-quitters in the intervention group. Additionally, quitters in the intervention group attended more than two times as many sessions as non-quitters in the intervention group [52,58-62,67]. Finally, in the study of Verweij on preventing weight gain by implementing an occupational health guideline, it was found that employees with higher attendance and satisfaction levels significantly reduced their waist circumference and body weight compared to employees with lower attendance and satisfaction levels [52,58-62,67].

## Discussion

The aims of this review were to (1) further our understanding of the quality of process evaluations alongside effect evaluations for WHPPs, (2) identify barriers/facilitators affecting implementation, and (3) explore the relationship between effectiveness and the implementation process.

Prior to discussing the main findings, it should be noted that only a small number of studies that evaluated the effectiveness of a WHPP included a process evaluation relating to the implementation of that WHPP. Of the 307 effect evaluations identified in this review, only twenty-two (7.2%) published an additional process evaluation. With respect to the first aim we can conclude that the quality of process evaluations alongside effect evaluations of WHPPs was generally poor to average and a systematic approach was lacking. This makes it difficult to draw firm conclusion about reliably identifying implementation barriers and/or facilitators (second aim) or about the relation between effectiveness and implementation (third aim). Murta et al. (2007) found the same difficulties in a systematic review. They concluded that process evaluations alongside workplace stress-management interventions were mainly poor to average, also making it difficult to identify reliable determinants of effective intervention implementation [22].

The process evaluations covered by our review lacked a theoretical basis, and the most frequently measured process components were dose delivered and dose received. This reflects the primary interest of researchers in actual intervention delivery and in participation levels (in other words, quantitative outcomes), rather than in how an intervention is delivered, the quality of delivery (fidelity), maintenance, or the reasons respondents participated or not. Possible explanations may be that researchers are more trained in quantitative methods of research and less in qualitative methods and analysis. Furthermore, good qualitative research takes a lot of time and energy and is therefore more expensive than quantitative research [75].

The included process evaluations tended to operationalise the measured process components in different ways, even when using the same framework for evaluation. For instance, four studies used Steckler and Linnan’s framework as a guideline for their process evaluation [51,57,58,69]. However, in some of these studies, actual attendance levels were placed under reach [51,57,58], whereas another study included attendance levels as part of the dose received [69]. The definitions for ‘reach’ and ‘dose received’ as defined by Steckler and Linnan are: ‘*proportion of intended target audience that participates in an intervention*’ and ‘*the extent to which participants actively engage with the intervention*’ respectively [14]. The different approaches to operationalisation in studies of these components could be explained by these somewhat ambiguous descriptions in Steckler and Linnan’s framework. What is the difference between ‘participating’ and ‘actively engaging with’ an intervention? We can assume that ‘participating in an intervention’ involves a focus on participation and non-participation regardless of the frequency, duration and intensity of participation and that these aspects may actually be taken into account when measuring active engagement with the intervention. Since several studies had different approaches to the operationalisation of process components, it was a challenge to translate the definitions used into general terms. We therefore adopted a working definition for each process component based on a previously published framework in order to interpret our findings [11]. For example, we defined reach as ‘*the proportion of the target audience that is aware of the intervention*’ and dose received as ‘*the proportion of participants that actually participates in the intervention*’, including frequency, duration and intensity [11]. We therefore distinguished between awareness and actual participation, the latter including level of participation. However, due to the operationalisation ambiguity in Steckler and Linnan’s framework, others might define these components, and therefore interpret the findings, differently. This limitation was also mentioned in a comparable review by Durlak and Dupre (2008), revealing that researchers still do not take the time and effort necessary to develop consensus about the best way to perform process evaluations in different settings [13].

Our review of the studies provided evidence of various barriers and/or facilitators that could influence the implementation process. However, partly as a result of the relatively low quality of the process evaluations, no systematic examination of barriers and/or facilitators affecting implementation was possible. Barriers and/or facilitators were often not systemically measured and only observed and documented on the basis of the researchers’ experience. This raises the question of how reliable the results of this review are with respect to the identified barriers and/or facilitators (Table 3). Since the barriers and/or facilitators identified in this review clearly overlap with other reviews focusing on these factors, we can conclude that our results should be reliable [10,12,13,22,24,25]. For example, Sangster-Gormley also identified ‘active management participation’ as a facilitator for implementation [25].

Another notable finding was that researchers evaluated barriers and/or facilitators mostly after implementation and only a few studied them during implementation. However, interventions are more likely to be successful if potential barriers and/or facilitators are assessed beforehand so they can be anticipated, facilitating implementation. Although most studies failed to observe barriers and/or facilitators systematically, some barriers and/or facilitators were more frequently reported in several studies. This suggests that these are important factors which researchers, practitioners and implementers need to take into account during a WHPP. In the category ‘characteristics of the socio-political context’, only one barrier (‘competitive business environment’) and one facilitator (‘compatibility of program with societal developments’) were reported in all twenty-two studies. This could suggest that this category is virtually disregarded, despite its importance for a thorough understanding of these socio-political factors prior to implementation, since the intended user of the intervention is part of an organization, which in turn is part of a wider environment [12]. However, it is also possible that intervention developers and researchers already anticipate on socio-political issues and try to take these into account throughout the development and implementation process.

Most barriers and/or facilitators were reported within the category ‘characteristics of the organization’. This is not surprising since most implementation research focusses at this level as this is essential for continuation. Moreover, the studies in this review are selected because they focus on organizations. Frequently mentioned implementation barriers were: ‘lack of resources’, ‘no fit of the intervention with organizational culture’ and ‘unbalanced or lack of manager support’. For example, when the intervention program requires a fitness center but there is no center nearby, this intervention is not the best fit with the company and other options, such as sports activities (possibly outdoors) in the immediate vicinity of the company, should be explored. Frequently mentioned organizational facilitators for WHPP implementation were: ‘organizations awareness of perceived benefits and relevance’; ‘good collaboration with all persons involved’; ‘strong and formal management support’; and ‘active management participation and engagement’. The researcher or implementer could work on these factors with the aim of optimizing implementation, for example by organizing manager meetings before the start of the intervention so that everyone is informed. However, another frequently mentioned facilitator for implementation was: ‘an organizational culture that emphasizes goal setting and supports social interaction’. Nevertheless, it is unclear what researchers and implementers can do when the organization culture does not fulfill this criterion before the start of the study, especially since organizational culture is a complex phenomenon which is not easily changed [76]. We suggest that the best way to deal with this ‘problem’ is to adapt the intervention where possible to ensure an optimal fit with the current organizational culture. In addition we would advise researchers and implementers to take the time to assess the organizational culture and include organizational determinants in their process evaluation [77].

Turning to the category ‘characteristics of the implementer’, the results of this review indicate that the most important facilitators for implementers are: ‘sufficient time’, ‘skills’, ‘knowledge’ and ‘competence’. When implementers experienced ‘planning difficulties’ or a ‘heavy workload’, this represented a barrier to implementation. However, these barriers can be overcome by making sure that the implementer receives enough support from stakeholders within the organization and enough administrative support. In the category ‘characteristics of the intervention program’, fifteen barriers and/or facilitators were reported. The three most frequently reported facilitators in this category were: ‘relative advantage’, ‘compatibility’ and ‘complexity’. These three factors are part of Rogers’ Diffusion of Innovations theory, and they are therefore known facilitating factors which could explain why researchers focused on these factors [78]. However, we also identified barriers relating to the intervention characteristics, including: ‘conflicting interests between worksite and interventions’ and ‘technical problems’ which are not part of a known theory. Fewest barriers and/or facilitators were reported in the category ‘characteristics of the participant’. The most commonly reported barrier to participation was ‘time constraints of participants’, suggesting that employers should give employees time off to participate in the interventions. This facilitator is also a reflection of the facilitator ‘management support and engagement’ which was mentioned earlier. Two frequently reported perceived barriers for participants were ‘work demands’ and ‘current workload’. However, as for many of the other found barriers, it is difficult to address these two factors. A possible suggestion to overcome these barriers could be that at onset of the study, employers should stress out that employees are allowed to participate under working hours and that if they experience difficulties in participating due to work demands and workload they should discuss this with their manager.

The reported barriers and/or facilitators in this review are comparable with the results of other reviews mentioned in this paper [10,12,24,25]. This suggests that the findings of this review are generalisable to other settings. It would be beneficial to explore all the barriers and/or facilitators listed here before implementation so that researchers and implementers can anticipate possible barriers, incorporate possible facilitators and perhaps adjust their program accordingly. A new Dutch instrument was proposed recently that makes it possible to map barriers and/or facilitators beforehand, and therefore possibly help to facilitate the actual implementation of WHPPs. It also serves as a monitoring instrument enabling program adjustments before and during implementation (TNO R10625; Fleuren et al. 2012).

Unfortunately, it was not possible to rank the 54 reported barriers and/or facilitators in order of importance because some determinants were only identified once. This could suggest that the factor was specific to the type of intervention. In addition, the relation between the 54 reported barriers and/or facilitators is unclear. This limitation was also experienced by Fleuren et al. (2004), showing that more research is needed into barriers and/or facilitators affecting the implementation process [12].

Despite the low quality of most included process evaluations, the findings of this review do suggest that higher levels of implementation are associated with better program outcomes. However, the few studies that investigated this association showed a narrow focus on implementation, mainly addressing the link between dose received and/or fidelity in relation to program outcomes. Whereas it is so important to use all aspects of process evaluations for the interpretation of the (lack of) effects of a program [18,19].

A limitation, which is inherent to writing a systematic review, is publication bias or studies being overlooked. In other words, our overview of the literature may not be complete. We tried to avoid this pitfall by selecting four different databases, both medical and psychological, using broad search terms and checking each lifestyle (i.e. physical activity, nutrition, smoking, alcohol use and relaxation) separately and by checking the references in the studies we included. Clearly, our decision to restrict our search to process evaluations in combination with effect evaluations in a (randomized) controlled trial design for WHPP has caused a number of process evaluations to fall out of scope for this review. Although we do acknowledge this, our aim was to gain insight into the relationship between implementation quality and effectiveness. Importantly, this is also a tendency that has been suggested by implementation journals, such as Implementation Science.

A best-evidence synthesis falls outside the scope of this review. Moreover, it could not be performed since we included only the studies which could be paired with a process evaluation. This means that not all known and relevant controlled trials in the field of WHPPs focusing on healthy lifestyle are included in this review.

## Conclusion

Given the fact that relatively few process evaluations were found by comparison with the high number of RCTs, and taking into account that not all process evaluations are published, it is safe to conclude that process evaluations are still not as high on the research agenda as effect evaluations. The importance of conducting an effect evaluation as well as a process evaluation is increasingly being advocated [22,79]. It does appear that lately more process evaluations are being conducted, but this review shows that they are in general of low to average quality due to the lack of a systematic approach.

Durlak and Dupre (2008) noted that “*implementation matters*” and “*science cannot study what it cannot measure accurately and cannot measure what is does not define*” [13]. Our findings support this observation, and moreover, suggest that we should be asking: what are we measuring, when we measure implementation at all? Without a standardized approach to the operationalisation of process evaluation components, it is difficult to faithfully replicate studies, identify implementation barriers and/or facilitators, or assess the methodological quality of process evaluations. This indicates that a general framework for process evaluations is required that researchers, implementers, reviewers and practitioners can use to evaluate and assess the quality of the implementation of a WHPP [80,81]. In order to create a general framework, it is essential that all relevant stakeholders subscribe to a consensus about the terminology and operationalisation of relevant process components by developing a taxonomy for process evaluations. This taxonomy could constitute a fundamental first step towards the standardization of process evaluations in multiple fields, and lead to program reproducibility [80]. Furthermore, it will allow researchers to compare and assess the quality of process evaluations, systematically identify implementation barriers and/or facilitators and possibly link implementation to program outcomes.

In short, this review demonstrates the need for a systematic approach to process evaluation as a way of improving WHPP implementation. In order to set the first steps into this direction, we suggest that future process evaluations need to apply the framework Wierenga et al. 2012 we proposed in the introduction of this review and should also use this framework to assess the methodological quality of the studies. This framework allows mapping the complete implementation process and takes into account determinants of implementation and provides the necessary explicit operationalization of each component [11].

## Abbreviations

WHPP: Worksite health promotion program.

## Competing interests

The author’s declare that they have no competing interests.

## Authors’ contributions

DW carried out the design, literature search, data extraction, quality analysis, data analysis and drafted the manuscript. LE and PvE participated in its design, data extraction, quality analysis, discussing the paper, providing methodological input, and helped to draft the manuscript. LE and SD were involved in the literature search. WvM and VH were involved in the design of the review and provided feedback of draft versions. All authors commented on the draft versions and read and approved the final manuscript.

## Pre-publication history

The pre-publication history for this paper can be accessed here:

http://www.biomedcentral.com/1471-2458/13/1190/prepub

## Supplementary Material

Additional file 1 Search strategies used for PubMed. Click here for file

Additional file 2 Characteristics of the studies included in this review. Click here for file
