# Supplementary material for: What is actually measured in process evaluations for worksite health promotion programs: a systematic review
Source: BMC Public Health. 2013 Dec 17;13:1190. doi: 10.1186/1471-2458-13-1190 (PMC3890539; doi:10.1186/1471-2458-13-1190)
Supplement: Additional file 1 — Search strategies used for PubMed. [file 1471-2458-13-1190-S1.docx]

**Search strategies used for PubMed**

| **Lifestyle behavior** | **Strategy** |
| --- | --- |
| Physical activity | ((Trial OR randomized controlled trial OR controlled trial) AND (Worker OR workers OR employee OR employees OR workforce OR worksite OR “work site” OR “work environment”) AND (health promotion OR worksite health promotion OR lifestyle intervention OR physical activity OR exercise OR physical fitness) AND (absenteeism OR sickness absence OR sick leave OR body mass index OR body weight OR lifestyle OR life style OR quality of life OR health OR health behavior OR blood pressure OR cholesterol level)) AND ((English OR Dutch[Language])) AND ("2000/01/01"[Date - Publication] : "2012/08/01"[Date - Publication])) |
| Nutrition | ((Trial OR randomized controlled trial OR controlled trial) AND (Worker OR workers OR employee OR employees OR workforce OR worksite OR “work site” OR “work environment”) AND (health promotion OR worksite health promotion OR lifestyle intervention OR Nutrition OR healthy eating OR food OR diet OR food services OR nutrition policy) AND (absenteeism OR sickness absence OR sick leave OR body mass index OR body weight OR lifestyle OR life style OR quality of life OR health OR health behavior OR blood pressure OR cholesterol level)) AND ((English OR Dutch[Language])) AND ("2000/01/01"[Date - Publication] : "2012/08/01"[Date - Publication])) |
| Smoking | ((Trial OR randomized controlled trial OR controlled trial) AND (Worker OR workers OR employee OR employees OR workforce OR worksite OR “work site” OR “work environment”) AND (health promotion OR worksite health promotion OR lifestyle intervention OR smoking OR Tobacco OR smoking cessation OR tobacco use) AND (absenteeism OR sickness absence OR sick leave OR body mass index OR body weight OR lifestyle OR life style OR quality of life OR health OR health behavior OR blood pressure OR cholesterol level OR stress)) AND ((English OR Dutch[Language])) AND ("2000/01/01"[Date - Publication] : "2012/08/01"[Date - Publication])) |
| Alcohol use | ((Trial OR randomized controlled trial OR controlled trial) AND (Worker OR workers OR employee OR employees OR workforce OR worksite OR “work site” OR “work environment”) AND (health promotion OR worksite health promotion OR lifestyle intervention OR Alcohol drinking OR Alcohol intake OR alcohol consumption) AND (absenteeism OR sickness absence OR sick leave OR body mass index OR body weight OR lifestyle OR life style OR quality of life OR health OR health behavior OR blood pressure OR cholesterol level OR stress)) AND ((English OR Dutch[Language])) AND ("2000/01/01"[Date - Publication] : "2012/08/01"[Date - Publication])) |
| Relaxation | ((Trial OR randomized controlled trial OR controlled trial) AND (Worker OR workers OR employee OR employees OR workforce OR worksite OR “work site” OR “work environment”) AND (health promotion OR worksite health promotion OR lifestyle intervention OR "stress management" OR "job stress" OR "work stress" OR "psychological stress" OR "life stress" OR "emotional stress" OR distress OR "mental suffering" OR "relaxation therapy" OR relaxation OR meditation OR mind-body OR "mind-body therapies" OR "leisure activities" OR mindfulness OR "mental healing" OR "exercise movement techniques" OR "breathing exercises" OR yoga OR "tai ji" OR pilates OR engagement OR vitality OR detachment) AND (absenteeism OR sickness absence OR sick leave OR body mass index OR body weight OR lifestyle OR life style OR quality of life OR health OR health behavior OR blood pressure OR cholesterol level OR stress OR relaxation)) AND ((English OR Dutch[Language])) AND ("2000/01/01"[Date - Publication] : "2012/08/01"[Date - Publication])) |

**Search strategies used for EMBASE**

| **Lifestyle behavior** | **Strategy** |
| --- | --- |
| Physical activity | 'controlled study'/exp OR 'randomized controlled trial'/exp OR 'intervention study'/exp AND ('worker'/exp OR 'employee'/exp OR 'personnel'/exp OR 'workplace'/exp OR 'work environment'/exp) AND ('health promotion'/exp OR 'lifestyle modification'/exp OR 'physical activity'/exp OR 'exercise'/exp OR 'fitness'/exp) AND ('absenteeism'/exp OR 'sickness absence'/exp OR 'medical leave'/exp OR 'body mass'/exp OR 'body weight'/exp OR 'lifestyle'/exp OR 'life style'/exp OR 'quality of life'/exp OR 'health'/exp OR 'health behavior'/exp OR 'blood pressure'/exp OR 'cholesterol blood level'/exp) AND ([dutch]/lim OR [english]/lim) AND [2000-2012]/py |
| Nutrition | 'controlled study'/exp OR 'randomized controlled trial'/exp OR 'intervention study'/exp AND ('worker'/exp OR 'employee'/exp OR 'personnel'/exp OR 'workplace'/exp OR 'work environment'/exp) AND ('health promotion'/exp OR 'lifestyle modification'/exp OR 'nutrition'/exp OR 'food'/exp OR 'diet'/exp OR 'catering service'/exp OR 'nutrition policy'/exp) AND ('absenteeism'/exp OR 'sickness absence'/exp OR 'medical leave'/exp OR 'body mass'/exp OR 'body weight'/exp OR 'lifestyle'/exp OR 'life style'/exp OR 'quality of life'/exp OR 'health'/exp OR 'health behavior'/exp OR 'blood pressure'/exp OR 'cholesterol blood level'/exp) AND ([dutch]/lim OR [english]/lim) AND [2000-2012]/py |
| Smoking | 'controlled study'/exp OR 'randomized controlled trial'/exp OR 'intervention study'/exp AND ('worker'/exp OR 'employee'/exp OR 'personnel'/exp OR 'workplace'/exp OR 'work environment'/exp) AND ('health promotion'/exp OR 'lifestyle modification'/exp OR 'smoking'/exp OR 'tobacco'/exp OR 'smoking cessation'/exp OR 'tobacco dependence'/exp) AND ('absenteeism'/exp OR 'sickness absence'/exp OR 'medical leave'/exp OR 'body mass'/exp OR 'body weight'/exp OR 'lifestyle'/exp OR 'life style'/exp OR 'quality of life'/exp OR 'health'/exp OR 'health behavior'/exp OR 'blood pressure'/exp OR 'cholesterol blood level'/exp OR 'stress'/exp) AND ([dutch]/lim OR [english]/lim) AND [2000-2012]/py |
| Alcohol use | 'controlled study'/exp OR 'randomized controlled trial'/exp OR 'intervention study'/exp AND ('worker'/exp OR 'employee'/exp OR 'personnel'/exp OR 'workplace'/exp OR 'work environment'/exp) AND ('health promotion'/exp OR 'lifestyle modification'/exp OR 'drinking behavior'/exp OR 'alcohol'/exp OR 'alcohol consumption'/exp) AND ('absenteeism'/exp OR 'sickness absence'/exp OR 'medical leave'/exp OR 'body mass'/exp OR 'body weight'/exp OR 'lifestyle'/exp OR 'life style'/exp OR 'quality of life'/exp OR 'health'/exp OR 'health behavior'/exp OR 'blood pressure'/exp OR 'cholesterol blood level'/exp OR 'stress'/exp) AND ([dutch]/lim OR [english]/lim) AND [2000-2012]/py |
| Relaxation | trial OR randomized AND controlled AND trial OR controlled AND trial AND ('worker'/exp OR workers OR 'employee'/exp OR employees OR workforce OR worksite OR 'work site' OR 'work environment'/exp) AND ('health'/exp AND promotion OR worksite AND 'health'/exp AND promotion OR 'lifestyle'/exp AND intervention OR 'stress management'/exp OR 'job stress'/exp OR 'work stress'/exp OR 'psychological stress'/exp OR 'life stress'/exp OR 'emotional stress'/exp OR 'distress'/exp OR 'mental suffering' OR 'relaxation therapy'/exp OR 'relaxation'/exp OR 'meditation'/exp OR 'mind body' OR 'mind-body therapies'/exp OR 'leisure activities'/exp OR mindfulness OR 'mental healing'/exp OR 'exercise movement techniques'/exp OR 'breathing exercises'/exp OR 'yoga'/exp OR 'tai ji'/exp OR 'pilates'/exp OR engagement OR vitality OR detachment) AND ('absenteeism'/exp OR 'sickness'/exp AND 'absence'/exp OR sick AND leave OR body AND 'mass'/exp AND index OR body AND 'weight'/exp OR 'lifestyle'/exp OR 'life'/exp AND style OR quality AND of AND 'life'/exp OR 'health'/exp AND 'behavior'/exp OR 'blood'/exp AND 'pressure'/exp OR 'cholesterol'/exp AND level OR 'stress'/exp OR 'relaxation'/exp) AND ([dutch]/lim OR [english]/lim) AND [2000-2012]/py |

**Search strategies used for PsycINFO**

| **Lifestyle behavior** | **Strategy** |
| --- | --- |
| Physical activity | (KW=(trial or randomized controlled trial or controlled trial) and (Worker or workers or employee or employees or workforce or worksite or 'work site' or 'work environment' or workplace or 'work place' or personnel or working conditions or business organizations) and (health promotion or worksite health promotion or lifestyle intervention or physical activity or exercise or physical fitness or 'active living') and (absenteeism OR sickness absence OR sick leave OR body mass index OR body weight OR lifestyle OR life style OR quality of life OR health OR health behavior OR blood pressure OR cholesterol level)) AND (Limiters - Published Date: 20000101-20120731; Language: Dutch, English; Document Type: Journal Article) |
| Nutrition | KW=(trial or randomized controlled trial or controlled trial) and (Worker or workers or employee or employees or workforce or worksite or 'work site' or 'work environment' or workplace or 'work place' or personnel or working conditions or business organizations) and (health promotion or worksite health promotion or lifestyle intervention or nutrition or food or diets or 'healthy eating' or 'food services' or 'nutrition policy') and (absenteeism OR sickness absence OR sick leave OR body mass index OR body weight OR lifestyle OR life style OR quality of life OR health OR health behavior OR blood pressure OR cholesterol level) AND (Limiters - Published Date: 20000101-20120731; Language: Dutch, English; Document Type: Journal Article) |
| Smoking | KW = (trial or randomized controlled trial or controlled trial) and (Worker or workers or employee or employees or workforce or worksite or 'work site' or 'work environment' or workplace or 'work place' or personnel or working conditions or business organizations) and (health promotion or worksite health promotion or lifestyle intervention or smoking OR Tobacco or Tobacco smoking or smoking cessation or tobacco use) and (absenteeism OR sickness absence OR sick leave OR body mass index OR body weight OR lifestyle OR life style OR quality of life OR health OR health behavior OR blood pressure OR cholesterol level OR stress) AND (Limiters - Published Date: 20000101-20120731; Language: Dutch, English; Document Type: Journal Article) |
| Alcohol use | KW = (trial or randomized controlled trial or controlled trial) and (Worker or workers or employee or employees or workforce or worksite or 'work site' or 'work environment' or workplace or 'work place' or personnel or working conditions or business organizations) and (health promotion or worksite health promotion or lifestyle intervention or 'drinking behavior' OR alcohol OR 'alcohol consumption' OR 'alcohol drinking') and (absenteeism OR sickness absence OR sick leave OR body mass index OR body weight OR lifestyle OR life style OR quality of life OR health OR health behavior OR blood pressure OR cholesterol level OR stress) AND (Limiters - Published Date: 20000101-20120731; Language: Dutch, English; Document Type: Journal Article) |
| Relaxation | KW = (trial or randomized controlled trial or controlled trial) and (Worker or workers or employee or employees or workforce or worksite or 'work site' or 'work environment' or workplace or 'work place' or personnel or working conditions or business organizations) and (health promotion or worksite health promotion or lifestyle intervention or "stress management" OR "job stress" OR "work stress" OR "psychological stress" OR "life stress" OR "emotional stress" OR distress OR "mental suffering" OR "relaxation therapy" OR relaxation OR meditation OR mind-body OR "mind-body therapies" OR "leisure activities" OR mindfulness OR "mental healing" OR "exercise movement techniques" OR "breathing exercises" OR yoga OR "tai ji" OR pilates OR engagement OR vitality OR detachment) and (absenteeism OR sickness absence OR sick leave OR body mass index OR body weight OR lifestyle OR life style OR quality of life OR health OR health behavior OR blood pressure OR cholesterol level OR stress OR relaxation)AND (Limiters - Published Date: 20000101-20120731; Language: Dutch, English; Document Type: Journal Article) |

**Search strategies used for Cochrane Central Register of Controlled trials**

| **Lifestyle behavior** | **Strategy** |
| --- | --- |
| Physical activity | (Trial or randomized controlled trial or controlled trial) and (Worker or workers or employee or employees or workforce or worksite or "work site" or "work environment") and (health promotion or worksite health promotion or lifestyle intervention or physical activity or exercise or physical fitness) and (absenteeism or sickness absence or sick leave or body mass index or body weight or lifestyle or life style or quality of life or health or health behavior or blood pressure or cholesterol level):ti,ab,kw from 2000 to 2012 (Word variations have been searched) |
| Nutrition | (Trial OR randomized controlled trial OR controlled trial) AND (Worker OR workers OR employee OR employees OR workforce OR worksite OR “work site” OR “work environment”) AND (health promotion OR worksite health promotion OR lifestyle intervention OR Nutrition OR healthy eating OR food OR diet OR food services OR nutrition policy) AND (absenteeism OR sickness absence OR sick leave OR body mass index OR body weight OR lifestyle OR life style OR quality of life OR health OR health behavior OR blood pressure OR cholesterol level) :ti,ab,kw from 2000 to 2012 (Word variations have been searched) |
| Smoking | (Trial OR randomized controlled trial OR controlled trial) AND (Worker OR workers OR employee OR employees OR workforce OR worksite OR “work site” OR “work environment”) AND (health promotion OR worksite health promotion OR lifestyle intervention OR smoking OR Tobacco OR smoking cessation OR tobacco use) AND (absenteeism OR sickness absence OR sick leave OR body mass index OR body weight OR lifestyle OR life style OR quality of life OR health OR health behavior OR blood pressure OR cholesterol level OR stress) :ti,ab,kw from 2000 to 2012 (Word variations have been searched) |
| Alcohol use | (Trial OR randomized controlled trial OR controlled trial) AND (Worker OR workers OR employee OR employees OR workforce OR worksite OR “work site” OR “work environment”) AND (health promotion OR worksite health promotion OR lifestyle intervention OR Alcohol drinking OR Alcohol intake OR alcohol consumption) AND (absenteeism OR sickness absence OR sick leave OR body mass index OR body weight OR lifestyle OR life style OR quality of life OR health OR health behavior OR blood pressure OR cholesterol level OR stress) :ti,ab,kw from 2000 to 2012 (Word variations have been searched) |
| Relaxation | (Trial OR randomized controlled trial OR controlled trial) AND (Worker OR workers OR employee OR employees OR workforce OR worksite OR “work site” OR “work environment”) AND (health promotion OR worksite health promotion OR lifestyle intervention OR "stress management" OR "job stress" OR "work stress" OR "psychological stress" OR "life stress" OR "emotional stress" OR distress OR "mental suffering" OR "relaxation therapy" OR relaxation OR meditation OR mind-body OR "mind-body therapies" OR "leisure activities" OR mindfulness OR "mental healing" OR "exercise movement techniques" OR "breathing exercises" OR yoga OR "tai ji" OR pilates OR engagement OR vitality OR detachment) AND (absenteeism OR sickness absence OR sick leave OR body mass index OR body weight OR lifestyle OR life style OR quality of life OR health OR health behavior OR blood pressure OR cholesterol level OR stress OR relaxation) :ti,ab,kw from 2000 to 2012 (Word variations have been searched) |
