# Supplementary material for: What is actually measured in process evaluations for worksite health promotion programs: a systematic review
Source: BMC Public Health. 2013 Dec 17;13:1190. doi: 10.1186/1471-2458-13-1190 (PMC3890539; doi:10.1186/1471-2458-13-1190)
Supplement: Additional file 2 — Characteristics of the studies included in this review. [file 1471-2458-13-1190-S2.docx]

Characteristics of the studies included in this review

| *General study information* | | *Process evaluation information* | |
| --- | --- | --- | --- |
| Reference | **Design effect evaluation:** Randomized controlled trial (RCT) or Controlled trial (CT)  **Type of company**  **Study population**  **Intervention content**  **Intervention goal**  **Proportion of affected primaryoutcomes** | **Data collection method of process evaluation(PE)**  **Timing of process evaluation (PE)**  **Process evaluation (PE) evaluation levels:** Macro, meso, micro  **Type of process evaluation (PE)**  **Effect of implementation on outcome measure:** Described or Not described | **Model used for evaluation**  **Measured process components:** Definition used in the study, - =not described |
| Driessen et al. (2010,2011) [24-26]  *Netherlands* | **Design effect evaluation:** RCT  **Type of company:** University, railway transportation, airline, steel  **Study population**: Blue and white collar  **Intervention content:**  Worksite participatory ergonomics program  **Intervention goal:** Prevent low back pain and neck pain  **Proportion of affected primaryoutcomes:** 2 of 14 | **Data collection method PE:**  Mixed methods  **Timing PE:** During and post-intervention  **PE evaluation levels:** Meso, Micro  **Type of PE:** Evaluation of interventions and implementation process  **Effect of implementation on outcome measure:** Not described | **Model used for evaluation:**  Adjusted version of Linnan and Steckler  **Measured process components:**   - *Recruitment:* intervention departments, working groups formed, working group members for training, response rate baseline questionnaire - *Reach:* worksite visits by trainer, attendance rates implementers, attendance rates of implementers in training - *Dose delivered:* perceived implementation of the intervention according to implementers - *Dose received:* perceived implementation of the intervention of the employees - *Fidelity:* Extent to which the intervention was delivered as intended - *Satisfaction:* of implementers about implementation process and intervention, and of employees about the intervention - *Context:* Perceived barriers and/or facilitators to implementation of intervention - *Maintenance:* - |
| Groeneveld et al. (2010, 2011)[27-29]  *Netherlands* | **Design effect evaluation:** RCT  **Type of company:** Construction  **Study population:** Blue collar at risk for CVD  **Intervention content:**  Individually based lifestyle intervention by means of motivational interviewing  **Intervention goal:** Change  physical activity, diet and smoking behavior  **Proportion of affected primaryoutcomes:** 8of 15^a^ | **Data collection method PE:** Mixed methods  **Timing PE:** Post-intervention  **PE evaluation levels:** Meso, Micro  **Type of PE:** Evaluation of interventions  **Effect of implementationon outcome measure:** Described for body weight | **Model used for evaluation:**  Not described  **Measured process components:**   - *Dose delivered:* Number of sessions performed by implementer and number of items discussed during session - *Dose received:* Number of sessions attended by participants - *Fidelity:* Adherence to intervention protocol by implementers and the quality of the intervention delivery - *Satisfaction:* Participants opinion on the competence and skills of the implementer. Implementers opinion on their own competence and skills on delivery of intervention and overall difficulty of delivering intervention - *Recruitment, Reach, Maintenance, Context:* - |
| French et al. (2010) [30, 31]  *United States* | **Design effect evaluation:** RCT  **Type of company:** Bus garages  **Study population:** Blue collar  **Intervention content:** Environmental worksite obesity prevention intervention  **Intervention goal:** Improve healthful food choices an physical activity levels  **Proportion of affected primaryoutcomes:** 4 of 20 | **Data collection method PE:** Quantitative  **Timing PE:** Post-intervention  **PE evaluation levels:** Micro  **Type of PE:** Evaluation of interventions  **Effect of implementationon outcome measure:**Not described | **Model used for evaluation:**  Not described  **Measured process components:**   - *Dose delivered:* Amount of interventions made available during the intervention period - *Dose received:* Participation rates of employees in intervention - *Recruitment, Reach, Fidelity, Satisfaction, Maintenance, Context:* - |
| Dishman & Wilson et al. (2009, 2010) [32, 33]  *United States* | **Design effect evaluation:** RCT  **Type of company:** Retail  **Study population:** White collar  **Intervention content:**  Social-ecologic intervention by personal and team goal-setting  **Intervention goal:** Increase leisure-time physical activity  **Proportion of affected primaryoutcomes:** 3 of 3 | **Data collection method PE:** Mixed methods  **Timing PE:** During and post-intervention  **PE evaluation levels:** Meso**,** Micro  **Type of PE:** Evaluation of interventions and implementation process  **Effect of implementationon outcome measure:**Described for physical activity levels | **Model used for evaluation:**  Durlak & Dupre  **Measured process components:**   - *Dose delivered:* Amount of intervention delivered - *Dose received:* Amount of intervention received by participants - *Fidelity:* Degree to which the intervention was implemented as planned - *Context:* Implementation barriers - *Recruitment, Reach, Satisfaction, Maintenance:* - |
| Yap et al. (2009, 2010) [34-36]  *United States* | **Design effect evaluation:** CT  **Type of company:** Manufacturing plant  **Study population:** Blue collar  **Intervention content:**  Tailored e-mail intervention  **Intervention goal:** Increase intentional physical activity  **Proportion of affected primaryoutcomes:** 2 of 2 | **Data collection method PE:** Qualitative  **Timing PE:** Post-intervention  **PE evaluation levels:**  Micro  **Type of PE:** Evaluation of interventions  **Effect of implementationon outcome measure:**Not described | **Model used for evaluation:**  Not described  **Measured process components:**   - *Dose received:* Use of intervention - *Satisfaction:* Satisfaction with the intervention components - *Recruitment, Reach, Dose delivered, Fidelity, Maintenance, Context:* - |
| Gilson et al. (2007, 2008) [37, 38]  *Unites Kingdom* | **Design effect evaluation:** RCT  **Type of company:** University  **Study population**: White collar  **Intervention content:**  Route and task-based walking **Intervention goal:** Improve work day step counts and health status  **Proportion of affected primaryoutcomes:** 1of 4 | **Data collection method PE:**  Qualitative  **Timing PE:** Post-intervention  **PE evaluation levels:** Micro  **Type of PE:** Evaluation of interventions and implementation process  **Effect of implementationon outcome measure:**Not described | **Model used for evaluation:**  Not described  **Measured process components:**   - *Recruitment:* Strategies to approach and attract employees - *Satisfaction:* Experiences during intervention - *Context:* Benefits/positives and problems/barriers associated with participation in intervention - *Reach, Dose delivered, Dose received, Fidelity, Maintenance:* - |
| Goetzel, DeJoy, Wilson et al. (2007, 2009, 2010, 2011) [39-43]  *United States* | **Design effect evaluation:** CT  **Type of company:** Manufacturing  **Study population**: Blue and white collar  **Intervention content:** Environmental weight management intervention  **Intervention goal:** Improve physical activity and healthy eating  **Proportion of affected primaryoutcomes:** 6 of 8 | **Data collection method PE:** Mixed methods  **Timing PE:** During and post-intervention  **PE evaluation levels:** Macro, Meso, Micro  **Type of PE:** Evaluation of interventions and implementation process  **Effect of implementation on outcome measure:**Not described | **Model used for evaluation:**  Integrative model  **Measured process components:**   - *Context:* Management support, job/task factors (physical and psychological demands of specific jobs), environmental factors (physical work environment and the social-organizational environment - *Recruitment, Reach, Dose delivered, Dose received, Fidelity, Satisfaction, Maintenance:* - |
| Lemon, Estabrook et al. (2010, 2011) [44, 45]  *United States* | **Design effect evaluation:** RCT  **Type of company:** Hospital  **Study population**: White collar  **Intervention:**  Ecological and environmental multilevel intervention  **Intervention goal:** Prevent weight gain by targeting healthy eating and physical activity  **Proportion of affected primaryoutcomes:** 0 of 1 | **Data collection method PE:** Mixed methods  **Timing PE:** Pre-, during, and post-intervention  **PE evaluation levels:** Macro, Meso, Micro  **Type of PE:** Evaluation of interventions and implementation process  **Effect of implementationon outcome measure:**Describedfor Body Mass Index | **Model used for evaluation:**  RE-AIM  **Measured process components:**   - *Recruitment:* Amount of recruited settings for participation and number that agreed to take part, representatives of participating settings (Adoption) - *Reach:* Participation rates, representativeness of participants, characteristics of participants and non-participants - *Dose delivered:* Implemented intervention components - *Dose received:* Use of intervention components - *Fidelity:* Extent to which the program was delivered as intended at program and individual level (Implementation) - *Maintenance:* Extent to which the behavior or policy of interest maintained over the long term at individual and institutional level - *Context:* Management support, job/task factors (physical and psychological demands of specific jobs), environmental factors (physical work environment and the social-organizational environment - *Effectiveness:* Behavioral outcomes and other outcomes including impact on quality of life - *Satisfaction:* - |
| Andersen et al. (2011) [46-48]  *Denmark* | **Design effect evaluation:** RCT  **Type of company:** Office  **Study population**: White collar  **Intervention content:**  Progressive resistance training (exercise program)  **Intervention goal:** Relieve neck/shoulder pain  **Proportion of affected primaryoutcomes:** 4 of 6 | **Data collection method PE:**  Quantitative  **Timing PE:** Pre-intervention  **PE evaluation levels:** Micro  **Type of PE:** Evaluation of interventions  **Effect of implementationon outcome measure:**Not described | **Model used for evaluation:**  Not described  **Measured process components:**   - *Context:* Prognostic factors for adherence to intervention - *Recruitment, Reach, Dose delivered, Dose received, Fidelity, Satisfaction, Maintenance:* - |
| Haukka, Pehkonen et al. (2009, 2010) [49, 50]  *Finland* | **Design effect evaluation:** RCT  **Type of company:** Municipal kitchens  **Study population**: Blue collar  **Intervention content:**  Participatory ergonomics intervention  **Intervention goal:** Decrease physical and mental workload  **Proportion of affected primaryoutcomes:** 0 of 8 | **Data collection method PE:** Mixed methods  **Timing PE:** Pre-, during, and post-intervention  **PE evaluation levels:** Meso**,** Micro  **Type of PE:** Evaluation of interventions and implementation process  **Effect of implementationon outcome measure:**Not described | **Model used for evaluation:**  Not described  **Measured process components:**   - *Dose delivered:* Time and visits made to worksite - *Dose received:* Participation rates - *Satisfaction:* General opinions regarding process, experiences of the intervention, - *Context:* Benefits or difficulties experiences of the project, facilitating factors for implementation, support and time needed for development, organization of work tasks during intervention - *Recruitment, Reach, Fidelity, Maintenance:-* |
| Sorensen, Hunt (2005, 2007) [51, 52]  *United States* | **Design effect evaluation:** RCT  **Type of company:** Manufacturing  **Study population**: Blue collar  **Intervention content:** Worksite intervention targeting fruit and vegetable consumption, red meat consumption, multivitamin use and physical activity  **Intervention goal:** Cancer prevention  **Proportion of affected primaryoutcomes:** 2 of 4 | **Data collection method PE:** Mixed methods  **Timing PE:** Post-intervention  **PE evaluation levels:** Meso**,** Micro  **Type of PE:**  Evaluation of interventions and implementation process  **Effect of implementationon outcome measure:**Not described | **Model used for evaluation:**  Not described  **Measured process components:**   - *Reach:* Program awareness - *Dose delivered:* Delivered interventions - *Dose received:* Program participation - *Context:* Management contacts regarding organizational changes, factors that influences implementation - *Recruitment, Fidelity, Satisfaction, Maintenance:* - |
| Beresford et al. (2000, 2001, 2010) [53-55]  *United States* | **Design effect evaluation:** RCT  **Type of company:** Hospitals, educational, governmental, professional agencies, construction, manufacturing, financial, retail, wholesale, service  **Study population**: Blue and white collar  **Intervention content:** Environmental and individual strategies  **Intervention goal:** Increase fruit and vegetable intake  **Proportion of affected primaryoutcomes:** 2 of 2^b^ | **Data collection method PE:** Mixed methods  **Timing PE:** Post-intervention  **PE evaluation levels:** Meso, Micro  **Type of PE:** Evaluation of interventions  **Effect of implementation on outcome measure:**Described for fruit and vegetable intake | **Model used for evaluation:**  Not described  **Measured process components:**   - *Reach:* Seeing or reading the intervention materials (use of intervention) - *Dose delivered:* Documentation of intervention activities by implementers - *Dose received:* Average intervention exposure per employee - *Context:* Worksite characteristics - *Recruitment, Fidelity, Satisfaction, Maintenance:* - |
| Sorensen, Hunt et al. (2007, 2010) [56, 57]  *United States* | **Design effect evaluation:** RCT  **Type of company:** Construction  **Study population**: Blue collar  **Intervention content:** Tailored telephone-delivered and mailed intervention  **Intervention goal:** Promote smoking cessation and increase fruit and vegetable consumption  **Proportion of affected primaryoutcomes:** 3 of 3 | **Data collection method PE:**  Qualitative  **Timing PE:** Post-intervention  **PE evaluation levels:** Micro  **Type of PE:**  Evaluation of interventions  **Effect of implementationon outcome measure:**Not described | **Model used for evaluation:**  Not described  **Measured process components:**   - *Dose delivered:* Number of interventions delivered - *Dose received:* Receiving intervention and materials - *Fidelity:* Extent to which the intervention was implemented - *Satisfaction:* Participant satisfaction with intervention - *Recruitment, Reach, Maintenance, Context:* - |
| Steenhuis et al. (2004) [58, 59]  *Netherlands* | **Design effect evaluation:** RCT  **Type of company:** Governmental and large companies  **Study population**: White collar  **Intervention:**  Environmental interventions including labeling of healthy foods, food supply program and educational program about healthy food  **Intervention goal:** Increase availability and knowledge of healthy foods  **Proportion of affected primaryoutcomes:** 1 of 3 | **Data collection method PE:**  Qualitative  **Timing PE:** Post-intervention  **PE evaluation levels:** Meso  **Type of PE:**  Evaluation of interventions and implementation process  **Effect of implementation on outcome measure:**Not described | **Model used for evaluation:**  Not described  **Measured process components:**   - *Satisfaction:* managers opinions on intervention - *Context:* difficulties with implementation, perceived benefits of intervention - *Recruitment, Reach, Dose delivered, Dose received, Fidelity, Maintenance:* - |
| Sorensen, Quintiliani et al. (2010) [60, 61]  *United States* | **Design effect evaluation:** CT  **Type of company:** Trucking terminals  **Study population**: Blue collar  **Intervention:** Telephone and print-delivered intervention  **Intervention goal:** Promote tobacco use cessation and improve weight management by healthy nutrition  **Proportion of affected primaryoutcomes:** 1 of 2 | **Data collection method PE:**  Quantitative  **Timing PE:** Post-intervention  **PE evaluation levels:** Meso**,** Micro  **Type of PE:** Evaluation of interventions  **Effect of implementationon outcome measure:**Described for smoking cessation | **Model used for evaluation:**  Not described  **Measured process components:**   - *Dose delivered:* Number of delivered interventions - *Dose received:* Perception of received intervention components and materials (Engagement in intervention components) - *Satisfaction:* Helpfulness of intervention - *Recruitment, Reach, Fidelity, Maintenance, Context:* - |
| Stoddard, Hunt et al. (2003, 2005) [62, 63]  *United States* | **Design effect evaluation:** RCT  **Type of company:** Retail  **Study population**: White collar (teens)  **Intervention content:** Behavioral tobacco control intervention  **Intervention goal:** Decrease smoking prevalence  **Proportion of affected main outcomes:** 0 of 2 | **Data collection method PE:**  Quantitative  **Timing PE:** During and post-intervention  **PE evaluation levels:** Meso**,** Micro  **Type of PE:**  Evaluation of interventions  **Effect of implementation on outcome measure:**Not described | **Model used for evaluation:**  Not described  **Measured process components:**   - *Dose delivered:* Amount of intervention delivered - *Dose received:* amount of intervention received, participation rates, motivation for participation in intervention, program awareness - *Recruitment, Reach, Fidelity, Satisfaction, Maintenance, Context:* - |
| Volpp, Kim et al. (2009, 2011) [64, 65]  *United States* | **Design effect evaluation:** RCT  **Type of company:** Multinational  **Study population**: Not described  **Intervention content:** Offeringfinancial incentives for smoking cessation  **Intervention goal:** Improve smoking cessation rates  **Proportion of affected primaryoutcomes:** 1 of 1 | **Data collection method PE:** Mixed methods  **Timing PE:** Post-intervention  **PE evaluation levels:** Micro  **Type of PE:** Evaluation of interventions  **Effect of implementationon outcome measure:**Described for smoking cessation | **Model used for evaluation:**  Not described  **Measured process components:**   - *Reach:* Program awareness - *Dose delivered:* Amount of intervention delivered - *Dose received:* amount of intervention received, participation rates, motivation for participation in intervention - *Recruitment, Fidelity, Satisfaction, Maintenance, Context:* - |
| Hasson et al. (2005, 2010) [66, 67]  *Sweden* | **Design effect evaluation:** RCT  **Type of company:** Information technology, media  **Study population**: White collar  **Intervention content:** Web-based health promotion and stress management training  **Intervention goal:** Decrease unwanted stress and promote health and recovery  **Proportion of affected primaryoutcomes:** 5 of 5 | **Data collection method PE:**  Qualitative  **Timing PE:** During  **PE evaluation levels:** Micro  **Type of PE:** Evaluation of interventions  **Effect of implementationon outcome measure:**Not described | **Model used for evaluation:**  Not described  **Measured process components:**   - *Dose received:* Frequency of replying to the screening tool - *Context:* Factors that determine use of program - *Recruitment, Reach, Dose delivered, Fidelity, Satisfaction, Maintenance: -* |
| Vermeer et al. (2011) [68, 69]  *Netherlands* | **Design effect evaluation:** RCT  **Type of company:** Worksite cafeterias of hospitals, universities, police departments, companies  **Study population**: White collar  **Intervention content:**  Environmental intervention introducing a small hot meal in addition to the existing size and a proportional pricing strategy in cafeterias  **Intervention goal:** Stimulate workers to replace their larger meal with a smaller meal  **Proportion of affected primaryoutcomes:** 0 of 2 | **Data collection method PE:** Mixed methods  **Timing PE:** Pre-, during and post-intervention  **PE evaluation levels:** Meso, Micro  **Type of PE:**  Evaluation of interventions  **Effect of implementationon outcome measure:**Not described | **Model used for evaluation:**  Baranowski & Stables and Rogers  **Measured process components:**   - *Recruitment:* Attracting agencies, implementers, or potential participants for corresponding parts of the program - *Dose received:* Extent to which participants view or read the materials that reach them - *Fidelity:* Extent to which the program is implemented as designed - *Maintenance:* Keeping participants involved in the programmatic and data collection, and extent to which participants continue to do any of the activities - *Context:* Aspects of the environment of an intervention - *Contamination:* extent to which participants received interventions from outside the program and the extent to which the control group receives the treatment - *Resources:* Materials or characteristics of agencies, implementers, or participants necessary to attain project goals - *Reach, Dose delivered, Satisfaction:-* |
| Strijk et al. (2011, 2012) [70-72]  *Netherlands* | **Design effect evaluation:** RCT  **Type of company:** Academic hospital  **Study population**: White collar  **Intervention content:** Vitality intervention consisting of weekly yoga and workout session, weekly unsupervised aerobic exercise, free fruit during sessions and three visits of personal vitality coach  **Intervention goal:** Improve lifestyle behaviors  **Proportion of affected primaryoutcomes:** 3 of 10 | **Data collection method PE:**  Quantitative  **Timing PE:** During and post-intervention  **PE evaluation levels:** Meso**,** Micro  **Type of PE:**  Evaluation of interventions  **Effect of implementationon outcome measure:**Not described | **Model used for evaluation:**  Steckler and Linnan  **Measured process components:**   - *Reach:* proportion of workers participating in intervention - *Dose delivered:* number of intervention components delivered by implementers - *Dose received:* Extent to which the workers were engaged in the intervention (attendance rates) - *Fidelity:* Extent to which the intervention was implemented as planned - *Satisfaction:* Workers attitude towards the intervention - *Context:*Organizational and environmental factors concerning the intervention - *Recruitment, Maintenance:-* |
| Verweij et al. (2011, 2012) [73-75]  *Netherlands* | **Design effect evaluation:** RCT  **Type of company:** University, bank, nursing home, spice factory, packaging company, municipality, consumer goods company  **Study population**: Blue and white collar  **Intervention content:** Occupational health guideline  **Intervention goal**: preventing weight gain by increasing PA, decreasing sedentary behavior, increasing fruit consumption or reducing energy intake derived from snacks  **Proportion of affected primaryoutcomes:** 2 of 6 | **Data collection method PE:** Mixed methods  **Timing PE:** During and post-intervention  **PE evaluation levels:** Meso**,** Micro  **Type of PE:**  Evaluation of interventions and implementation process  **Effect of implementationon outcome measure:**Described for body weight and waist circumference | **Model used for evaluation:**  Steckler and Linnan  **Measured process components:**   - *Recruitment:* Sources and procedures used to approach and attract potential participants, the number of randomized OPs, and the number of employees that filled out the baseline questionnaire - *Reach:* Number of employees who attended the counseling sessions, reason for missed counseling sessions and the percentage of drop-outs including reason - *Dose delivered:* The number of intervention materials or components actually delivered by OPs, and the duration and form of the counseling sessions - *Dose received:* The extent to which participants use materials, resources, or techniques recommended by program - *Fidelity:* The extent to which the intervention was delivered as planned: if OPs adhered to the guideline and adequately performed behavior change counseling - *Satisfaction:* Participants attitudes toward the content, use and limitations of the guideline (OP), or toward the intervention, materials and OP (employee) - *Context:*Organizational characteristics that affect intervention implementation, including physical, social, political, and economic features - *Maintenance:* - |
| Jorgensen et al.(2011, 2012) [76-78]  *Denmark* | **Design effect evaluation:** RCT  **Type of company:** Hospitals, cleaning companies, large business with in-house cleaning services  **Study population**: Blue collar  **Intervention content:** Physical coordination training  **Intervention goal:** Improve physical coordination training or cognitive behavioral resources  **Proportion of affected primaryoutcomes:** 3 of 6 | **Data collection method PE:**  Quantitative  **Timing PE:** During intervention  **PE evaluation levels:** Meso  **Type of PE:** Evaluation of interventions  **Effect of implementationon outcome measure:**Not described | **Model used for evaluation:**  Adapted version Steckler & Linnan  **Measured process components:**   - *Recruitment:* Procedures used to approach and attract participants - *Dose delivered:* intervention delivered - *Dose received:* adherence to intervention (attendance rates) - *Fidelity:* Quality of intervention delivery - *Context:* Unanticipated events at the work place - *Reach, Satisfaction, Maintenance:* - |

RCT: Randomized controlled trial; CT: Controlled trial; PE: process evaluation; Macro level: company/management; Meso level: Implementer; Micro: employee
^a^ 3 outcomes were significant at short and long term, 3 outcomes only at short term, 2 outcomes only at long term
^b^ both outcomes were significantly improved at short term, however they were not statistically significant at long term but still improved in favor of intervention group
